# Atrial fibrillation signals associated with overactive bladder drugs across JADER and FAERS: disproportionality and time-to-onset analyses

**DOI:** 10.3389/fphar.2025.1700587

**Published:** 2026-01-08

**Authors:** Kyosuke Nagura, Satoko Watanabe, Taro Watanabe, Hidenori Sagara

**Affiliations:** 1 Division of Medical Safety Science, Faculty of Pharmaceutical Sciences, Sanyo-Onoda City University, Sanyo-Onoda, Japan; 2 Department of Pharmacy, Yamaguchi Prefectural Grand Medical Center, Hofu, Japan; 3 Division of Medical Safety Science, Graduate School of Pharmaceutical Sciences, Sanyo-Onoda City University, Sanyo-Onoda, Japan

**Keywords:** atrial fibrillation, mirabegron, solifenacin, disproportionality analysis, FDA adverse event reporting system, Japanese adverse drug event report, pharmacovigilance, time-to-onset

## Abstract

**Introduction:**

Overactive bladder (OAB) drugs are widely prescribed, yet the occurrence of atrial fibrillation (AF) after treatment initiation remains poorly characterized.

**Methods:**

We evaluated reports of AF associated with OAB medications using two spontaneous reporting systems (SRSs): the Japanese Adverse Drug Event Report (JADER) database and the U.S. FDA Adverse Event Reporting System (FAERS). We screened eight agents and assessed signals using three disproportionality metrics: the reporting odds ratio (ROR), proportional reporting ratio (PRR), and Bayesian confidence propagation neural network (BCPNN). For drugs showing signals in both databases, we conducted stratified analyses by sex, age, and number of concomitant medications, and evaluated time-to-onset (TTO) using Weibull modeling.

**Results:**

Consistent AF signals were identified for solifenacin succinate and mirabegron, whereas other agents did not meet the prespecified criteria. Solifenacin met the criteria in women and older adults in both JADER and FAERS. Mirabegron met the criteria across multiple strata in both datasets, indicating cross-stratum reproducibility. TTO was right‐skewed, with most reports occurring within one year of initiation. Exploratory Weibull modeling, based on limited numbers of date‐complete reports, suggested a wear-out pattern for solifenacin in JADER and an early pattern in FAERS, while mirabegron showed a random pattern in JADER and an early pattern in FAERS. These failure‐type patterns should therefore be interpreted cautiously.

**Discussion:**

These findings are hypothesis-generating, given the limitations of SRSs, such as underreporting, missing dates, and unknown exposure—and they reflect reporting patterns rather than causal risk. They outline strata and early treatment periods that may warrant clinical attention and help prioritize pharmacovigilance and targeted hypothesis‐driven evaluation in routine OAB care.

## 1 Introduction

Clinicians sometimes observe sudden arrhythmias, particularly atrial fibrillation (AF), after initiating overactive bladder (OAB) therapy in older adults, yet systematic evidence remains limited. Antimuscarinics (AMs) and β3-adrenergic receptor agonists (β3-ARAs) can influence autonomic tone and heart rate (van der Hooft et al., 2004; Nitti et al., 2014), raising questions about potential cardiovascular (CV) associations. AF is unpredictable and may result in severe outcomes (Dilaveris and Kennedy, 2017). Beyond its role as a major cause of cardioembolic stroke, AF has also been associated with cognitive decline and dementia, including cases in patients without clinically overt stroke (de Bruijn et al., 2015). In addition, AF itself is an independent risk factor for all-cause mortality and a major driver of unplanned hospitalization, with population-based meta-analyses suggesting roughly 1.5–2-fold higher death rates among individuals with AF than among those without AF (Odutayo et al., 2016). Thus, many patients with AF require long-term oral anticoagulants to prevent stroke, which can be particularly challenging to balance against bleeding risk in older adults. Although clinical trials and observational studies of OAB therapies have generally shown no clear association with AF (Chapple et al., 2013; Margulis et al., 2018; Tadrous et al., 2019), their design limitations and short follow-up periods can miss sudden or silent AF (Dilaveris and Kennedy, 2017). Moreover, the heavy reliance on tolterodine as a comparator in these studies restricts drug-specific safety estimates for individual OAB agents (Rosa et al., 2016; Margulis et al., 2018; Tadrous et al., 2019).

Spontaneous reporting systems (SRSs) complement trials by avoiding restrictive eligibility criteria and enabling longer observation periods. Although limited in detecting silent AF, these data reflect real-world care and can reveal early associations between drugs and rare, severe adverse events, including AF (Kasliwal, 2012). To our knowledge, no SRS-based study has provided a focused, multidrug, multi-database assessment of OAB drugs and AF. In the FDA Adverse Event Reporting System (FAERS), a screening analysis of mirabegron detected an AF signal using the reporting odds ratio (ROR) and Bayesian confidence propagation neural network (BCPNN), but the signal was not examined in detail (Wang et al., 2024).

SRS research now extends beyond early signal detection to include stratified analyses and time-to-onset (TTO) evaluations based on event and drug initiation dates (Sandberg et al., 2020; Liu et al., 2024; Lou et al., 2025). These methods help characterize when the reports occur and which patient characteristics are associated with reporting. The major systems include VigiBase [World Health Organization (WHO)], FAERS [U.S. Food and Drug Administration (FDA)], and JADER [Pharmaceuticals and Medical Devices Agency (PMDA)] (Pharmaceuticals and Medical Devices Agency PMDA, 2015; U.S. Food and Drug Administration, 2024; Uppsala Monitoring Centre, 2025). Cross-database comparisons can enhance detection; when signals replicate across systems, their reproducibility and generalizability as hypotheses are strengthened (Candore et al., 2015). Conversely, conflicting signals across systems may reflect system-specific characteristics—reporting practices, clinical settings and case mix, and population structure (e.g., racial/ethnic composition)—and can, therefore, be informative for understanding the strengths and blind spots of each SRS rather than indicating mutually exclusive findings.

In this study, using two SRSs—JADER and FAERS—and placing high priority on the replication of signals across systems, we evaluated AF reports associated with eight OAB drugs through disproportionality screening, stratified analyses, and TTO assessment. Our objective was to characterize reporting patterns and assess their reproducibility across databases, rather than to estimate incidence or risk.

## 2 Methods

### 2.1 Database source

**FIGURE 1:**
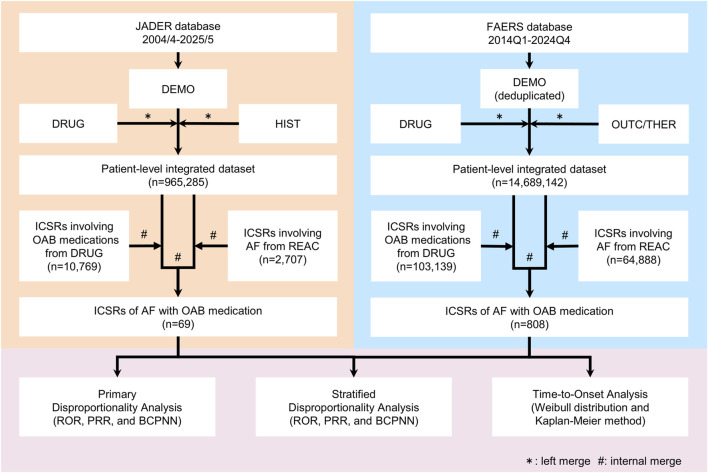
Study workflow. Data preprocessing (deduplication and integration), construction of a patient-level dataset, case selection, and analyses—including disproportionality screening, subgroup stratification, and time-to-onset (TTO)—were performed in JADER and FAERS. Abbreviations: AF, atrial fibrillation; BCPNN, Bayesian confidence propagation neural network; DEMO, demographic table; DRUG, drug table; FAERS, FDA Adverse Event Reporting System; HIST, medical history table; ICSR, individual case safety report; JADER, Japanese Adverse Drug Event Report database; OAB, overactive bladder; OUTC, outcome table; PRR, proportional reporting ratio; REAC, adverse event table; ROR, reporting odds ratio; THER, therapy table; TTO, time-to-onset.

Figure 1 outlines the workflow. We analyzed JADER (April 2004–May 2025) and FAERS (Q1 2014–Q4 2024), which were downloaded from the PMDA and FDA, respectively. For JADER, we constructed a patient-level integrated dataset (PLID) by merging DEMO with DRUG and HIST using the identification number. For FAERS, we de-duplicated DEMO by retaining the record with the highest *caseversion* for each *caseid* and then merged the de-duplicated DEMO with DRUG, OUTC, and/or THER via *primaryid* to create the PLID. This approach ensured that only the latest individual case safety reports (ICSRs) were analyzed. Table linkage varied by analysis: stratified analyses used DEMO + DRUG + OUTC, whereas TTO used DEMO + DRUG + THER, and both were keyed to *primaryid*.

### 2.2 Identification of AF reports associated with OAB medications

Eight OAB drugs approved in Japan—oxybutynin chloride, propiverine chloride, solifenacin succinate, imidafenacin, tolterodine tartrate, fesoterodine fumarate, mirabegron, and vibegron—were selected to carry out a direct comparison between JADER and FAERS. Drug names were standardized using Python. Records were extracted from DRUG and linked to the PLID using the case identifier (JADER identification number; FAERS *primaryid*). To minimize selection bias, we included entries where the drug role was classified as suspected, concomitant, or interacting. AF records were extracted using the MedDRA Preferred Term “atrial fibrillation” (PT 10003658; MedDRA/J v28.0) (Brown et al., 1999). AF records were extracted from REAC and linked to the PLID using the same identifier. The OAB and AF datasets were then linked to generate the final analysis dataset of AF reports associated with OAB drugs.

### 2.3 Disproportionality analysis

A 2 × 2 table was used to screen for AF signals for each drug (Table 1; cell counts *n*_*11*_, *n*_*12*_, *n*_*21*_, and *n*_*22*_ as defined therein). We computed three disproportionality measures: ROR, proportional reporting ratio (PRR), and BCPNN [information component (IC)] (Orre et al., 2000; Evans et al., 2001; van Puijenbroek et al., 2002; Sakaeda et al., 2013). Disproportionality analyses were implemented as hypothesis-generating procedures, consistent with good pharmacovigilance practice outlined by CIOMS Working Group VIII (Council for International Organizations of Medical Sciences CIOMS, 2010). Thresholds and reporting conventions were pre-specified to enhance transparency and reproducibility. To ensure minimum statistical stability, strata with fewer than three AF reports for the target drug (*n*_*11*_ < 3) were excluded from ROR, PRR, and BCPNN calculations.

**TABLE 1:** 2 × 2 table for AF signal detection with OAB drugs.

​	With AF	Without AF	Total
OAB drugs	*n*_*11*_	*n*_*12*_	*n*_*1+*_
Non-OAB drugs	*n*_*21*_	*n*_*22*_	*n*_*2+*_
Totals	*n*_*+1*_	*n*_*+2*_	*n*_*++*_

Cell definitions: *n*_*11*_, AF reports with OAB drugs; *n*_*12*_, non-AF reports with OAB drugs; *n*_*21*_, AF reports with non-OAB drugs; *n*_*22*_, non-AF reports with non-OAB drugs. Row totals (*n*_*1+*_, *n*_*2+*_), column totals (*n*_*+1*_, *n*_*+2*_), and overall total *n*_*++*_. This table forms the basis for ROR, PRR, and BCPNN calculations.

Abbreviations: AF, atrial fibrillation; OAB, overactive bladder; ROR, reporting odds ratio; PRR, proportional reporting ratio; BCPNN, Bayesian confidence propagation neural network.

Signals were defined as follows: for ROR, ROR_025_ > 1 together with two-sided Fisher’s exact *p* < 0.05; for PRR, a stringent threshold of PRR_025_ > 2 with χ^2^ > 4 (stricter than the conventional PRR_025_ > 1; adopted to corroborate ROR-based signals under a more conservative criterion and to mitigate spurious positives; to our knowledge, this combination is rarely described in prior SRS screening protocols); and for BCPNN, IC_025_ > 0, with strength categorized as weak (0 < IC_025_ < 1.5), medium (1.5 ≤ IC_025_ < 3.0), or strong (IC_025_ ≥ 3.0) (Sun et al., 2019). ROR_025_, PRR_025_, and IC_025_ denote the lower bounds of the 95% confidence intervals (CIs).

**FIGURE 2:**
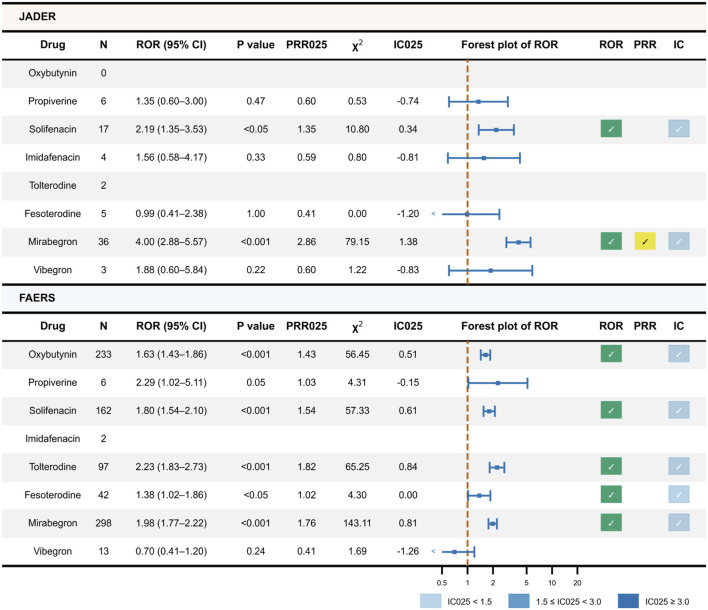
Primary disproportionality screening of AF reports associated with OAB drugs in JADER and FAERS. Top: JADER; bottom: FAERS. From left to right, the table shows drug, N, ROR (95% CI) with *p*-value, PRR_025_ and χ^2^, IC_025_, a forest plot of ROR (dashed line at ROR = 1), and check marks indicating whether the predefined thresholds were met for ROR, PRR, and IC (Methods 2.3). IC strength is color-coded: light blue for 0 < IC_025_ < 1.5, blue for 1.5 ≤ IC_025_ < 3.0, and dark blue for IC_025_ ≥ 3.0. Source data for Figure 2 are available in Supplementary Data S2. Abbreviations: AF, atrial fibrillation; OAB, overactive bladder; JADER, Japanese Adverse Drug Event Report database; FAERS, FDA Adverse Event Reporting System; *N*, number of reports; CI, confidence interval; ROR, reporting odds ratio; PRR, proportional reporting ratio; IC, information component; PRR_025_/IC_025_, lower 95% CI bounds for PRR/IC.

Exact formulas, CI calculations, and BCPNN prior settings are provided in Supplementary Data S1 (Sections S1.2–S1.4). All cell counts and metrics underlying the primary screening are provided as source data in Supplementary Data S2 (Figure 2).

### 2.4 Stratified disproportionality analysis

Stratified analyses were conducted for drugs with AF signals replicated in both JADER and FAERS (Section 2.3). Three variables were used: sex, age group, and count of concomitant medications. Sex was categorized as male or female. The age range was from 20 to 99 years and was categorized as 20–69 years (non-elderly) and 70–99 years (elderly) (Kim, 2023). Concomitant medication count was estimated using the maximum drug sequence number recorded per case; this approximates the number of medications reported but does not ensure simultaneous use. Polypharmacy was defined as ≥5 medications, and non-polypharmacy was defined as <5 (Masnoon et al., 2017). Records missing any required stratum were excluded during data cleaning.

**FIGURE 3:**
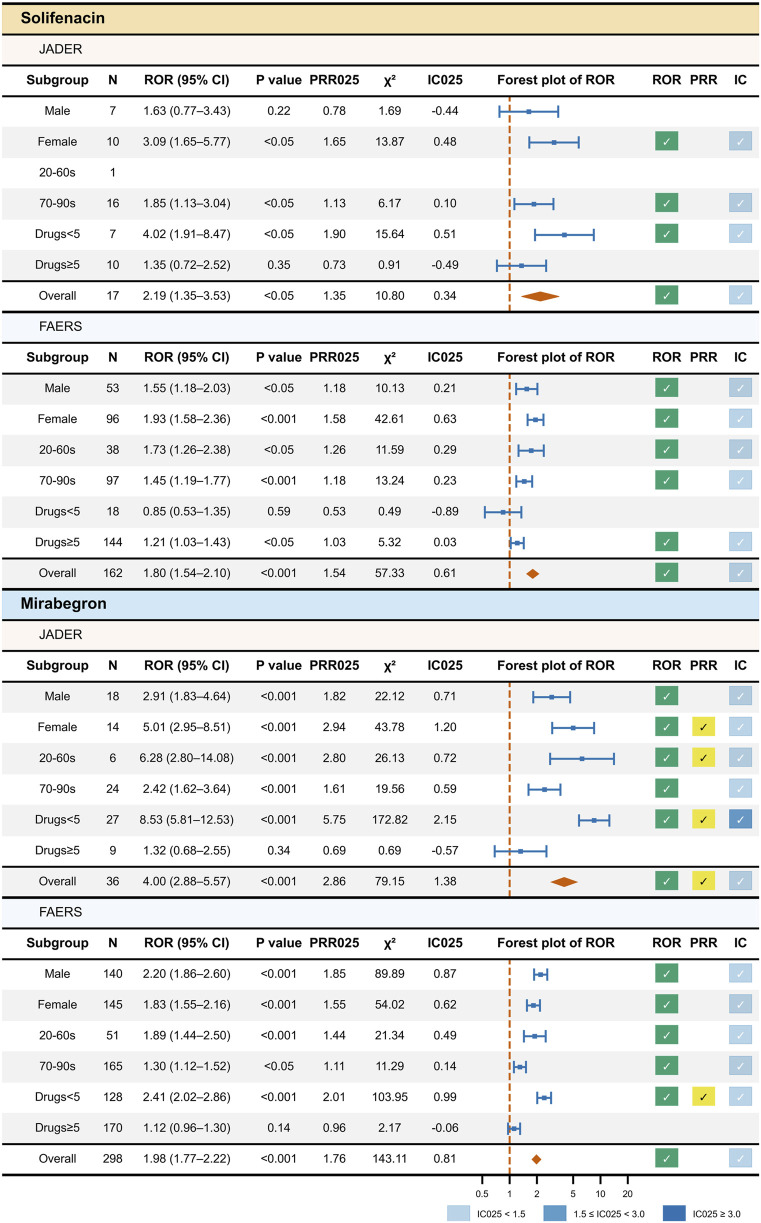
Stratified disproportionality screening of AF reports for solifenacin and mirabegron in JADER and FAERS. Top to bottom panels: solifenacin in JADER; solifenacin in FAERS; mirabegron in JADER; mirabegron in FAERS. Results are stratified by sex, age group, and concomitant medication count. From left to right, the table shows the stratum, *N*, ROR (95% CI) with *p*-value, PRR_025_ and χ^2^, IC_025_, a forest plot of ROR (dashed line at ROR = 1), and check marks indicating whether predefined thresholds were met for ROR, PRR, and IC (Methods 2.3). IC strength is color-coded: light blue for 0 < IC_025_ < 1.5, blue for 1.5 ≤ IC_025_ < 3.0, and dark blue for IC_025_ ≥ 3.0. Source data for Figure 3 are available in Supplementary Data S3. Abbreviations: AF, atrial fibrillation; JADER, Japanese Adverse Drug Event Report database; FAERS, FDA Adverse Event Reporting System; N, number of reports; CI, confidence interval; ROR, reporting odds ratio; PRR, proportional reporting ratio; IC, information component; PRR_025_/IC_025_, lower 95% confidence bounds for PRR/IC; Drugs <5/≥5, concomitant medication count strata.

To aid interpretation, volcano plots were generated for each drug, with the x-axis representing ln(ROR) and the y-axis representing −log10(p). Each point corresponds to a stratum of the chosen variables. The per-stratum source data used to render the stratified forest plot (Figure 3) are provided in Supplementary Data S3.

### 2.5 Time-to-onset analysis

TTO for AF with OAB drugs was defined as the number of days from drug initiation to event onset, with 1 day added to avoid zero-day intervals (Nakao et al., 2017; Ohyama et al., 2018). In JADER, the initiation and event dates were obtained from DRUG and REAC, respectively; in FAERS, they were obtained from THER (initiation) and DEMO (event). If multiple records of the same drug exposure and AF occurred within a single report, the earliest initiation and onset date pair was used (Hasegawa et al., 2020). TTO was summarized using the median and interquartile range (IQR).

For Weibull analysis, the shape parameter (β) was estimated using maximum likelihood, and 95% CIs for β were obtained via nonparametric bootstrap with 10,000 resamples (Estadística et al., 2018). The failure type was classified based on the 95% CI of β; it was classified as wear-out if the lower bound >1, random if the CI included 1, and early if the upper bound <1. According to technical documentation for PASS software (NCSS, 2022), simulation studies of the Weibull shape parameter suggest that approximately 70–80 failure events are needed to obtain a 90% CI with a relative half-width of approximately 15%. Given the small number of date-complete cases (n = 5–77), these results are considered exploratory.

To aid interpretation, we also created descriptive visuals, including boxplots, histograms, and Kaplan–Meier (KM) curves. These plots were not used for formal hypothesis testing and are intended solely to illustrate the reporting patterns. No *ad hoc* truncation was applied in the supplementary visualization; the full-window KM curves are presented in Supplementary Figure S4.

### 2.6 Tools for analysis

**FIGURE 4:**
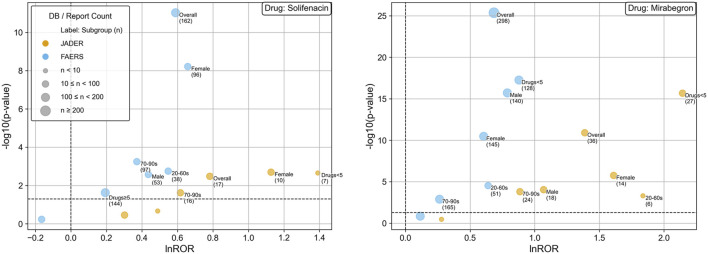
Panels: Left, solifenacin; right mirabegron. Colors: JADER (orange), FAERS (blue). Points: each dot represents one stratum (sex, age, concomitant medication count); where present, an “Overall (N)” point summarizes the pre-stratified result for that database and drug. Axes: x = ln(ROR); y = –log10(p). Point size reflects the number of reports. Reference lines: vertical at ln(ROR) = 0 (no disproportionality) and horizontal at –log10(p) = 1.30 (p = 0.05). These plots illustrate reporting patterns from the analysis pipeline outputs and are not used for hypothesis testing. Abbreviations: ROR, reporting odds ratio; ln, natural logarithm; p, p-value; JADER, Japanese Adverse Drug Event Report database; FAERS, FDA Adverse Event Reporting System. (Age bands on the plots are abbreviated but correspond to 20–69 years and 70–99 years, as defined in Methods.)

**FIGURE 5:**
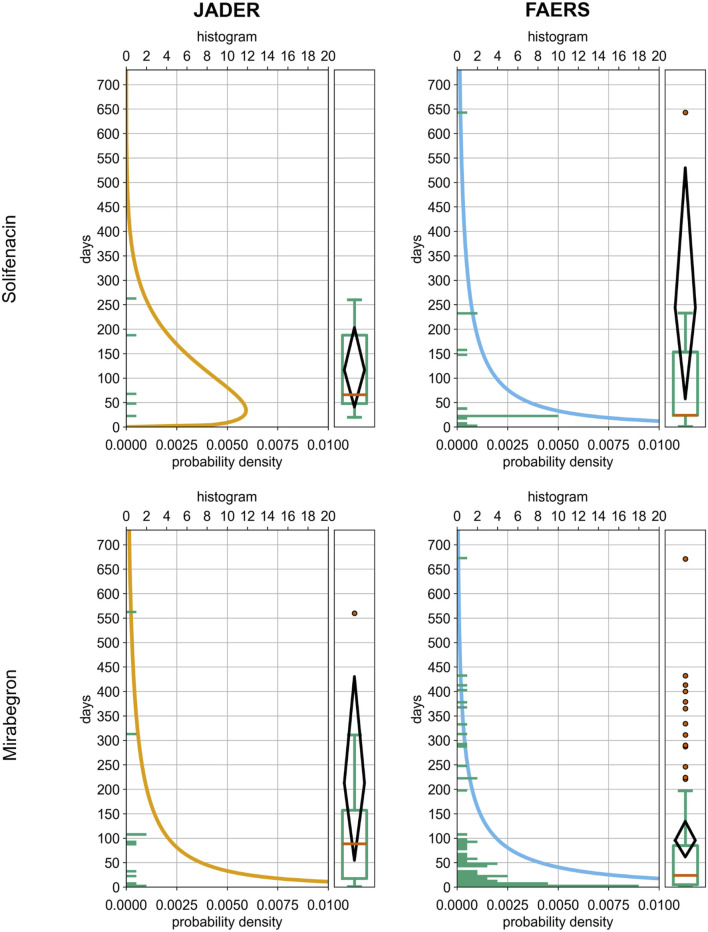
Panels: Upper left, solifenacin in JADER; upper right, solifenacin in FAERS; lower left, mirabegron in JADER; lower right, mirabegron in FAERS. Elements: a Weibull probability density curve, a histogram of report counts (5‐day bins), and a boxplot summarizing TTO within 2 years (0–730 days) after drug initiation. Axes: x = probability density; y β days. Panel titles indicate the database; colors follow a consistent plotting palette. These visuals are descriptive and support the interpretation of failure types derived from the Weibull shape parameter β. Abbreviations: AF, atrial fibrillation; TTO, time-to‐onset; JADER, Japanese Adverse Drug Event Report database; FAERS, FDA Adverse Event Reporting System; β, Weibull shape parameter.

**FIGURE 6:**
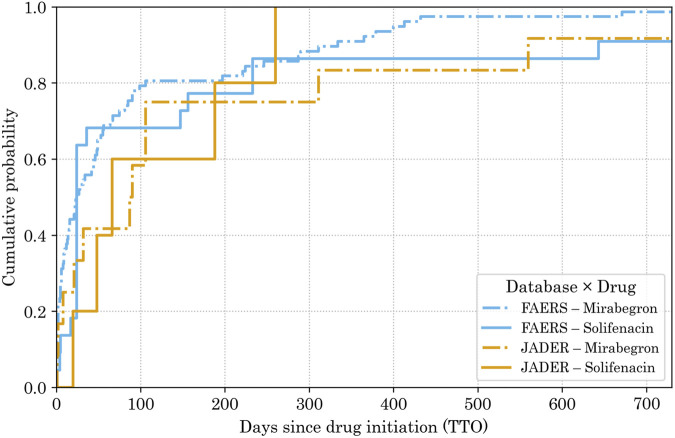
Cumulative proportion of atrial fibrillation (AF) reports associated with solifenacin and mirabegron in JADER and FAERS. KM curves were generated using TTO within 2 years (0–730 days) after drug initiation. Axes: x = TTO (days); y = cumulative proportion of reports (%). Line styles: solid (solifenacin), dash–dot (mirabegron). Colors: JADER (orange), FAERS (blue). This descriptive visualization highlights early clustering of AF onset and is not intended for hypothesis testing. An untruncated version covering the full observation window is provided as Supplementary Figure S4. Abbreviations: AF, atrial fibrillation; KM, Kaplan–Meier; TTO, time-to-onset; JADER, Japanese Adverse Drug Event Report database; FAERS, FDA Adverse Event Reporting System.

Analyses were conducted using MSIP (v1.10.1; NTT DATA Mathematical Systems, Inc.) and Python (v3.13). Plots in Figures 2–6 were generated using study‐specific Python scripts to ensure consistency between the analysis outputs and the manuscript figures.

### 2.7 Sensitivity analyses (Excel-only)

To assess robustness, the disproportionality analyses (Sections 2.3 and 2.4) were repeated under three alternative specifications. The metrics, CIs, and signal definitions followed those stated in Section 2.3, unless stated otherwise. Results are provided as an editable spreadsheet in Supplementary Data S5 (Excel); no figures were generated.

1. Conventional PRR threshold: PRR_025_ > 1 with χ^2^ > 4 (compared with the main threshold of PRR_025_ > 2).
2. Primary suspected only: the DRUG table was restricted to the primary suspected records in both databases; other drug roles were excluded.
3. History/indication exclusion: reports with prior AF in HIST (JADER) or AF listed as indication in INDI (FAERS) were excluded.

For each scenario, we report, by drug, database, and stratum (overall, sex, age 20–69 years or 70–99 years, and concomitant medication count <5 or ≥5): *n*_*11*_–*n*_*22*_, ROR (95% CI) with Fisher’s *p*, PRR (95% CI) with χ^2^, and IC (95% CI), along with indicators of whether the pre-specified thresholds were met (met_ROR, met_PRR, and met_IC). IC strength is also categorized as weak, medium, or strong, as defined in Section 2.3.

### 2.8 Negative control (Excel-only)

As a negative control, vibegron was included in each scenario above and processed using the same data handling, metrics, and thresholds as the primary drugs. Negative control rows appear alongside solifenacin and mirabegron in Supplementary Data S5 (Excel) for side-by-side comparison. No figures were generated.

## 3 Results

During the study periods, 69 and 808 AF reports associated with OAB drugs were identified in JADER and FAERS, respectively. These occurred among 965,285 and 14,689,142 total ICSRs, respectively.

### 3.1 Primary disproportionality analysis

Figure 2 summarizes the primary screening. In JADER, solifenacin met the signal criteria based on ROR and weak IC, while mirabegron met the criteria based on ROR, PRR, and weak IC. The other OAB drugs did not meet the predefined thresholds. In FAERS, oxybutynin, solifenacin, tolterodine, fesoterodine, and mirabegron met the criterion based on ROR and weak IC. Only solifenacin and mirabegron showed replicated signals across databases; these drugs were, therefore, advanced to stratified and TTO analyses.

### 3.2 Stratified disproportionality analysis

Figure 3 summarizes stratified screening by sex, age, and concomitant medication count.

For solifenacin, signals met the criteria in JADER among women, individuals aged 70–99 years, and cases with <5 concomitant medications (by ROR and weak IC). In FAERS, signals met the criteria across both sexes, individuals aged 20–69 years and 70–99 years, and cases with ≥5 concomitant medications (by ROR and weak IC).

For mirabegron, signals met the criteria in JADER among men and individuals aged 70–99 years (by ROR and weak IC) and among women, individuals aged 20–69 years, and cases with <5 concomitant medications (by ROR, PRR, and weak-to-medium IC). In FAERS, signals met the criteria across both sexes, individuals aged 20–69 years and 70–99 years (by ROR and weak IC), and cases with <5 concomitant medications (by ROR, PRR, and weak IC).

Volcano plots for solifenacin and mirabegron are shown in Figure 4. Points from JADER tend to lie further to the right (higher ln(ROR)) than those from FAERS. These visuals reflect reporting patterns rather than incidence, and cross-database magnitudes should be interpreted with caution.

### 3.3 Time-to-onset analysis

Table 2 summarizes TTO among date-complete reports. The median (IQR) TTO values were as follows: for solifenacin, JADER 66 (48–188) days and FAERS 24 (24–154) days; for mirabegron, JADER 89 (18–157) days and FAERS 24 (5–85) days.

**TABLE 2:** Time-to-onset analysis of AF reports with solifenacin and mirabegron in JADER and FAERS.

Drug	Time-to-onset (days)		Weibull distribution	
	Case (*n*)	Median (IQR)	β (95% CI)	Type
JADER				
Solifenacin	5	66 (48–188)	1.49 (1.42–69.52)	Wear-out failure
Mirabegron	12	89 (18–157)	0.57 (0.44–1.01)	Random failure
FAERS				
Solifenacin	22	24 (24–154)	0.53 (0.44–0.83)	Early failure
Mirabegron	77	24 (5–85)	0.56 (0.50–0.65)	Early failure

This table summarizes date-complete cases. For each drug and database, it shows cases (*n*), median TTO with IQR (days), Weibull shape parameter (β) with bootstrap 95% CI (10,000 resamples), and the failure type derived from β. Failure-type rules: β < 1, early; β ≈ 1, random; β > 1, wear-out. Values are descriptive and exploratory.

Abbreviations: AF, atrial fibrillation; TTO, time-to-onset; IQR, interquartile range; CI, confidence interval; JADER, Japanese Adverse Drug Event Report database; FAERS, FDA Adverse Event Reporting System; β, Weibull shape parameter.

β suggested a wear-out failure pattern for solifenacin in JADER (β = 1.49, 95% CI 1.42–69.52) and an early-failure pattern in FAERS (β = 0.53, 95% CI 0.44–0.83). For mirabegron, the pattern was random in JADER (β = 0.57, 95% CI 0.44–1.01) and early-failure in FAERS (β = 0.56, 95% CI 0.50–0.65). Given the small number of date-complete AF reports in each DB–drug stratum (n = 5–77), these failure-type patterns are considered exploratory. However, the early-failure pattern for mirabegron in FAERS, based on 77 reports, is comparatively more stable than that derived from ≤22 cases.

To focus on early-onset dynamics, Figures 5, 6 are limited to ≤2 years; for Figure 6, the full observation window is shown in Supplementary Figure S4. Figure 5 shows right-skewed onset distributions, with ≥80% of reports occurring within 1 year of treatment initiation. Consistent with this pattern, 92.2% (71/77) of FAERS mirabegron reports occurred within 365 days. KM curves (Figure 6) corroborate this early clustering and reveal broadly similar temporal patterns between JADER and FAERS.

### 3.4 Sensitivity analyses (Excel-only)

Across the three pre-specified sensitivity specifications (Methods 2.7), estimates and signal calls were consistent with those in the primary analysis. The only qualitative difference was that in JADER, the elderly stratum (70–99 years) for solifenacin did not meet any signal criterion when reports with pre-existing AF (primary disease/comorbidity) were excluded.

### 3.5 Negative control (Excel-only)

As a negative control drug, vibegron was processed identically to the primary analyses across all sensitivity scenarios (Methods 2.8). Vibegron did not meet any signal criterion in either database or any stratum. Negative control rows are provided alongside solifenacin and mirabegron in Supplementary Data S5 (Excel) for side-by-side comparison; no figures were generated.

## 4 Discussion

### 4.1 Overview of key findings

Across two SRS (JADER and FAERS), solifenacin and mirabegron met the pre-specified disproportionality criteria for AF, whereas other OAB drugs did not. These signals replicated across databases.

Patterns differed by database in stratified screening. For solifenacin, signals met the criteria in JADER among women, patients aged 70–99 years, and those with <5 concomitant medications, whereas in FAERS, they met the criteria across both sexes, individuals aged 20–69 years and 70–99 years, and those with ≥5 concomitant medications. For mirabegron, in JADER, signals met the criteria among men and those aged 70–99 years (by ROR and weak IC) and among women, patients aged 20–69 years, and those with <5 medications (by ROR and PRR and weak-to-medium IC). In FAERS, mirabegron met the criteria across both sexes, both age groups, and those with <5 medications.

TTO findings were descriptive. Weibull shape parameters suggested a wear-out failure pattern for solifenacin in JADER and an early-failure pattern in FAERS, whereas for mirabegron, they were random in JADER and early-onset in FAERS. Onset distributions were right-skewed, with most reports occurring within 1 year of treatment initiation. However, these apparent failure-type patterns were derived from small numbers of date-complete AF reports in each DB–drug stratum (n = 5–77) and are best regarded as exploratory timing tendencies rather than definitive classifications, even though the early-failure pattern for mirabegron in FAERS, based on 77 reports, is comparatively more stable than those derived from ≤22 cases.

These findings are hypothesis-generating; because of the inherent limitations of SRS data, disproportionality metrics do not estimate incidence or risk. Signal magnitudes reflect the reporting frequency and may be influenced by under-/over-reporting, confounding by indication, co-prescribing, stimulated reporting, and duplicate or miscoded records. Apparent stratum differences from stratified screening may, therefore, reflect prescribing or reporting patterns rather than pharmacologic effects and should be interpreted cautiously. Finally, TTO analyses were exploratory given the limited number of date-complete ICSRs, and precise estimation of the Weibull shape parameters generally requires several dozen to approximately 70–80 events; accordingly, the Weibull parameters should be understood as descriptive rather than inferential.

### 4.2 Comparison with previous studies

Randomized trials and large observational studies generally report no clear association between OAB therapy and AF, often concluding cardiovascular safety comparable to that of tolterodine tartrate (Rosa et al., 2016; Margulis et al., 2018; Tadrous et al., 2019). However, restrictive eligibility criteria, limited follow-up, and single-comparator designs may miss short, transient, or silent AF.

In contrast, our analyses of SRS replicated AF signals for solifenacin and mirabegron in both JADER and FAERS. Stratified and TTO analyses indicate where and when the reports cluster. These results reflect reporting patterns rather than true incidence or risk.

SRSs cannot reliably capture silent AF because such events may remain unrecognized and unreported. Nevertheless, their broad and heterogeneous coverage, combined with long reporting periods, can help identify patterns in rare or hard-to-detect events. These differences underscore the need for targeted evaluations using study designs capable of estimating risk.

### 4.3 Comparison and positioning with previous SRS studies

A prior FAERS-based screening of mirabegron reported an AF signal but did not examine AF in detail across drugs, strata, or timing (Wang et al., 2024). To our knowledge, no disproportionality analysis of solifenacin has jointly used JADER and FAERS.

Within this context, our study provides an AF-focused, multidrug, multi-database SRS evaluation that integrates stratified and TTO analyses. The aim is to generate a hypothesis and characterize the reporting patterns rather than risk estimation.

### 4.4 Identification of specific OAB medications associated with AF

Among the OAB drugs screened, only solifenacin and mirabegron met the AF signal criteria in both JADER and FAERS; the other drugs did not. Importantly, crossing the AF signal threshold reflects reporting intensity rather than causality, and results should be interpreted with caution. This pattern can suggest that the effects are drug-level rather than class-wide for AMs or β3-ARAs. Within β3-ARAs, mirabegron exhibited replicated signals, whereas vibegron did not reach the threshold in either database. A pharmacologic context may be relevant. Previous studies indicate greater β3 selectivity/potency for vibegron, whereas mirabegron exhibits slight β1/β2 activity (Brucker et al., 2022). These external data are provided for context only; mechanisms cannot be inferred from SRS findings.

### 4.5 Patient background characteristics requiring attention

Stratified analyses mapped where AF signals for solifenacin and mirabegron met the criteria across sex, age, and concomitant medication count (Figure 3).

For solifenacin, signals met the criteria in JADER among women, patients aged 70–99 years, and those with <5 concomitant medications; in FAERS, signals met the criteria across both sexes, in age groups 20–69 years and 70–99 years, and those with ≥5 concomitant medications. In several strata, detections differed between the two SRSs and, in some instances, the directions appeared conflicting. First, in JADER, the signal replicated among women but not among men. This pattern may be consistent with underlying epidemiology and utilization: OAB is reported more frequently in women than in men (Zhang et al., 2025), and in Japan, solifenacin is prescribed approximately 60% to women (Ministry of Health, Labour and Welfare [MHLW], 2025); women also tend to have longer QTc and are more susceptible to drug-induced QT prolongation (Vicente et al., 2020), which has been linked to AF (Ding et al., 2024). Together with the relatively small number of date-complete ICSRs in some strata, these factors may have contributed to the woman-only signal in JADER. Second, in JADER, the signal was not detected in the 20–69-year stratum. This may reflect age-related utilization—OAB prevalence increases from approximately the sixth decade (Zhang et al., 2025), and prescription of solifenacin in Japan is approximately 85% in those aged 70–99 years (MHLW, 2025)—and sparse data (the 20–69-year stratum contained only one ICSR), limiting statistical stability. At a minimum, these descriptive findings suggest that heightened attention may be necessary for women and older patients, while recognizing that these are signals of reporting and not of incidence or risk.

For mirabegron, in JADER, signals met the criteria among men and patients aged 70–99 years (by ROR and weak IC) and among women, patients aged 20–69 years, and those with <5 concomitant medications (by ROR, PRR, and weak-to-medium IC). In FAERS, mirabegron met the criteria across both sexes, both age groups, and those with <5 concomitant medications.

As with the primary disproportionality analyses, these stratified patterns are constrained by the inherent limitations of SRSs—the absence of exposure denominators, incomplete covariates, and multiple reporting biases (e.g., under-/over-reporting, confounding by indication, co-prescribing, stimulated reporting, and duplicate/miscoded records). In addition, JADER contains fewer ICSRs than FAERS in many strata, which can further destabilize signal estimates. Consequently, apparent stratum differences may reflect prescribing or reporting patterns rather than pharmacological effects and should be interpreted with caution.

Volcano plots (Figure 4) show that many JADER points shifted further right on ln(ROR) compared with FAERS points. This rightward shift may reflect differences in drug utilization, case mix, and reporting practices, and it could also be influenced by population differences, including ethnic composition. However, cross-database magnitudes are not directly comparable as SRSs lack denominators and are subject to multiple biases (Tubert-Bitter et al., 2016; Fusaroli et al., 2024).

### 4.6 Duration of use and reporting patterns

TTO results are summarized in Table 2 and Figures 5, 6. Onset distributions were right-skewed, with most date-complete reports occurring within 1 year of treatment initiation in both databases.

Weibull shape parameters differed by database. For solifenacin, the patterns suggested a wear-out failure type in JADER (β = 1.49, 95% CI 1.42–69.52; n = 5) and an early-failure type in FAERS (β = 0.53, 95% CI 0.44–0.83). For mirabegron, patterns were random in JADER (β = 0.57, 95% CI 0.44–1.01; n = 12) and indicated early failure in FAERS (β = 0.56, 95% CI 0.50–0.65; n = 77). Given these small counts and the fact that precise estimation of Weibull shape parameters generally requires several dozen to approximately 70–80 events, these apparent failure-type patterns should be regarded as exploratory timing tendencies rather than definitive classifications. Even the early-failure pattern for mirabegron in FAERS is best interpreted as hypothesis-generating, although it is comparatively more stable because it is based on 77 reports.

External context is broadly consistent with these tendencies—early clustering after initiation aligns with sympathomimetic effects described for mirabegron (Mo et al., 2017)—but these links remain speculative and do not imply causality.

### 4.7 Relationship with polypharmacy

We used the number of concomitant medications as a surrogate for comorbidity and overall health status. Although polypharmacy is often associated with drug-related adverse events (Davies et al., 2020), mirabegron met the AF signal criteria in the non-polypharmacy strata (<5 medications) in both databases, with gaps in the higher medication strata. Moreover, these signals were supported not only by ROR and IC but also by PRR under a more stringent threshold than the conventional PRR_025_ >1, which was adopted to corroborate ROR-based findings and mitigate false positives. This pattern may reflect pharmacologic factors or dilution/masking from multiple co-reported drugs and events in the ≥5 strata. In practice, AF monitoring should not be limited to patients with high medication counts.

### 4.8 Use of SRS databases and signal detection methods

We analyzed two independent SRSs (JADER and FAERS), consistent with recommendations to enhance generalizability. Differences in population and reporting across SRSs suggest that cross-database replication can help identify signals with broader relevance (Candore et al., 2015).

To balance sensitivity and specificity, we used ROR, PRR, and BCPNN. ROR is a frequentist measure that is widely used and suitable for broad screening; it is commonly applied in analyses of JADER (Sakaeda et al., 2013). PRR, adopted by the UK MHRA (Evans et al., 2001), compares event proportions. We applied a stringent threshold using the lower 95% confidence bound (PRR_025_), which is stricter than the conventional criterion, to corroborate ROR-based findings under a more conservative criterion and to mitigate false positives. BCPNN, used in VigiBase, is more robust at low counts and provides three IC strength levels that aid in interpretation (Bate et al., 1998; Sun et al., 2019). Combining these metrics offers a practical framework for SRS-based safety evaluation. Taken together, a multi-database approach combined with multiple algorithms (ROR, PRR, and BCPNN) aligns with CIOMS WG VIII recommendations for signal detection and consistency assessment (Council for International Organizations of Medical Sciences (CIOMS), 2010).

### 4.9 Limitations of SRS studies

SRS data are susceptible to underreporting, duplicate submissions, and notoriety- or stimulus-driven reporting, which can inflate counts following publicity or safety communications (de Boissieu et al., 2014; Alomar et al., 2020). Differences in labeling and clinical practice across regions can also influence reporter attribution and affect whether a drug is coded as suspected. For example, solifenacin lists AF in the Japanese label but not in the U.S. label (Astellas Pharma USInc, 2022; Pharmaceuticals and Medical Devices Agency, 2023). In a sensitivity analysis restricted to primary suspected drugs, solifenacin met the criteria in JADER but not in FAERS (Supplementary Data S5, Excel). Furthermore, because denominators and exposure time are unknown, ROR, PRR, and IC reflect relative reporting rather than incidence or risk, making quantitative comparisons across databases inappropriate (Tubert-Bitter et al., 2016; Fusaroli et al., 2024). In addition, this SRS-only analysis did not include an independent external validation cohort, which may limit the generalizability of these findings and highlights the need for confirmation through other data sources and study designs.

### 4.10 Methodological and disease-specific limitations

We used the maximum drug sequence number recorded per case as a proxy for polypharmacy, which may not fully capture concurrent exposure. Information on the starting dates, duration, and dose was limited, and the number of date-complete samples was small, introducing uncertainty in TTO estimates. For example, if a starting date reflects the prescription date rather than the first dose, TTO may be overestimated. Outcome coding may also misclassify AF under related events, such as stroke or heart failure. Importantly, silent AF requires confirmation by electrocardiography (ECG) and is difficult to ascertain, so episodes may remain unrecognized in SRSs (Kerr et al., 1996; Dilaveris and Kennedy, 2017; Joglar et al., 2024). Although continuous monitoring could improve detection, incorporating such an assessment into traditional cohort studies poses feasibility and ethical challenges. In future work, external validation of these SRS-derived signals will require study designs that can more completely ascertain asymptomatic and paroxysmal AF. One pragmatic option would be a large health system or claims-based cohorts that link OAB prescribing data with scalable rhythm monitoring strategies, including intermittent or continuous ECG patches and validated consumer wearable devices (e.g., smartwatch- or smartphone-based photoplethysmography and single-lead ECG). Large-scale studies such as the Apple Heart Study and subsequent validation work suggest that these technologies can detect AF with higher sensitivity and specificity than reference ECG, while reducing the burden of repeated 24-h Holter monitoring (Perez et al., 2019; Gill et al., 2022; Park and Bae, 2025). Such external validation cohorts could test whether the reporting patterns observed here correspond to increased incidents of AF in routine OAB care and help define monitoring strategies that are both clinically informative and acceptable to patients.

## 5 Conclusion

Using two SRSs (JADER and FAERS), we evaluated AF reporting for eight OAB drugs through disproportionality screening, stratified analyses, and TTO assessment. Solifenacin and mirabegron met the pre-specified AF signal criteria in both databases. Reporting patterns varied by drug and stratum: solifenacin met the criteria more frequently in women and older adults, whereas mirabegron met the criteria across multiple strata.

Most date-complete reports occurred within approximately 1 year of treatment initiation. These cross-database patterns, including the exploratory timing tendencies suggested by the Weibull analyses, can help prioritize pharmacovigilance and guide targeted evaluation in designs that are capable of estimating risk, including those that clarify when cardiac monitoring is most informative.

In clinical practice, these hypothesis-generating signals suggest that clinicians may pay particular attention to the patient characteristics highlighted here and maintain vigilance for cardiovascular parameters (e.g., blood pressure and heart rate), obtaining electrocardiography when clinically indicated—particularly during early treatment. However, the failure-type and timing patterns observed here are based on limited numbers of date-complete reports and should be viewed as tentative signals rather than definitive guidance. These points are intended to inform pharmacovigilance priorities rather than to imply incidence or causality.

The findings provide a foundation for future prospective studies and external validation cohorts aimed at confirming and further characterizing these associations and at more precisely defining any high-risk periods or patient groups.

## Data Availability

Publicly available datasets were analyzed in this study. The JADER dataset is available from the Pharmaceuticals and Medical Devices Agency (PMDA) (https://www.pmda.go.jp/safety/info-services/drugs/adr-info/suspected-adr/0004.html), and the FAERS Quarterly Data Extract files are available from the U.S. Food and Drug Administration (FDA) (https://www.fda.gov/drugs/fdas-adverse-event-reporting-system-faers/fda-adverse-event-reporting-system-faers-latest-quarterly-data-files). Further details are provided in the article and Supplementary Material.
