# Supplementary material for: Atrial fibrillation signals associated with overactive bladder drugs across JADER and FAERS: disproportionality and time-to-onset analyses
Source: Front Pharmacol. 2026 Jan 8;16:1700587. doi: 10.3389/fphar.2025.1700587 (PMC12823915; doi:10.3389/fphar.2025.1700587)
Supplement: Supplementary file 1 [file DataSheet4.pdf]

Supplementary Material

Purpose. This data sheet provides the full computational details that accompany Methods Section 2.3 in the main text. Unless otherwise noted, ln(·) denotes the natural logarithm. Formula numbering (S1–S3) is used for cross-reference from the article.

1 S1.1 2×2 table and notation

For each drug, counts were organized as follows (Table 1 in the main text):

- $n11$: AF reports with the target OAB drug
- $n12$: non-AF reports with the target OAB drug
- $n21$: AF reports with non-OAB drugs
- $n22$: non-AF reports with non-OAB drugs

Totals:

- $n1+ = n11 + n12$; $n2+ = n21 + n22$
- $n+1 = n11 + n21$; $n+2 = n12 + n22$
- $n{+}{+} = n11 + n12 + n21 + n22$

Minimum stability rule. Strata with $n11 < 3$ were excluded from ROR/PRR/BCPNN calculations.

2 S1.2 Reporting odds ratio (ROR)

Equation (S1):

$$ROR = (n11/n21) / (n12/n22) \quad (S1)$$

95% CI (log-scale normal approximation):

$$\ln(ROR) \pm 1.96 \times \mathrm{sqrt}(1/n11 + 1/n12 + 1/n21 + 1/n22) \Rightarrow 95\%\ CI = \exp(\cdot)$$

Significance reporting. Two-sided Fisher's exact p values were computed and used for the ROR-based signal criterion.

Signal definition (prespecified). $ROR025 > 1$ with two-sided Fisher's exact $p < 0.05$; ROR025 is the lower 95% CI bound.

3 S1.3 Proportional reporting ratio (PRR)

Equation (S2):

$$PRR = (n11/n1+) / (n21/n2+) \quad (S2)$$

95% CI:

$\ln(PRR) \pm 1.96 \times \mathrm{sqrt}(1/n11 - 1/n1+ + 1/n21 - 1/n2+) \Rightarrow$ 95% CI = exp(·)

Chi-square. Pearson χ^2 (df = 1) was computed.

Signal definition (stringent; prespecified). PRR025 > 2 and $\chi^2 > 4$ (more stringent than the conventional PRR025 > 1).

4 S1.4 Bayesian confidence propagation neural network (BCPNN)

Information component (IC) with Jeffreys-type priors.

Posterior mean:

$$E(IC11) = \log2(((n11+\gamma11)(n++ + \alpha)(n++ + \beta)) / ((n++ + \gamma)(n1+ + \alpha1)(n+1 + \beta1))) \quad (S3)$$

Posterior variance:

$$V(IC11) = (1/\ln2)^2 [(n++ - n11 + \gamma - \gamma11) / ((n11 + \gamma11)(1 + n++ + \gamma))$$

$$+ (n++ - n1+ + \alpha - \alpha1) / ((n1+ + \alpha1)(1 + n++ + \alpha))$$

$$+ (n++ - n+1 + \beta - \beta1) / ((n+1 + \beta1)(1 + n++ + \beta))]$$

Hyperparameters:

$\gamma = \gamma11 \times ((n++ + \alpha)(n++ + \beta)) / ((n1+ + \alpha1)(n+1 + \beta1))$; $\gamma11 = 1$; $\alpha1 = \beta1 = 1$; $\alpha = \beta = 2$.

95% interval (±2 approximation):

$IC025 = E(IC11) - 2\sqrt{V(IC11)}$, $IC975 = E(IC11) + 2\sqrt{V(IC11)}$.

Signal definition (prespecified). IC025 > 0.

IC strength categories. weak $0 < IC025 < 1.5$, medium $1.5 \leq IC025 < 3.0$, strong $IC025 \geq 3.0$.

5 S1.5 Interpretation and reporting conventions

• ROR, PRR, and IC are hypothesis-generating measures of reporting disproportionality, not incidence or risk.

• Thresholds and reporting conventions (ROR025/PRR025/IC025) were prespecified in line with good pharmacovigilance practice.

• All symbols and cell definitions follow Table 1 in the main text.
