# Supplementary material for: Atrial fibrillation signals associated with overactive bladder drugs across JADER and FAERS: disproportionality and time-to-onset analyses
Source: Front Pharmacol. 2026 Jan 8;16:1700587. doi: 10.3389/fphar.2025.1700587 (PMC12823915; doi:10.3389/fphar.2025.1700587)
Supplement: Supplementary file 3 [file Image1.pdf]

Supplementary Material

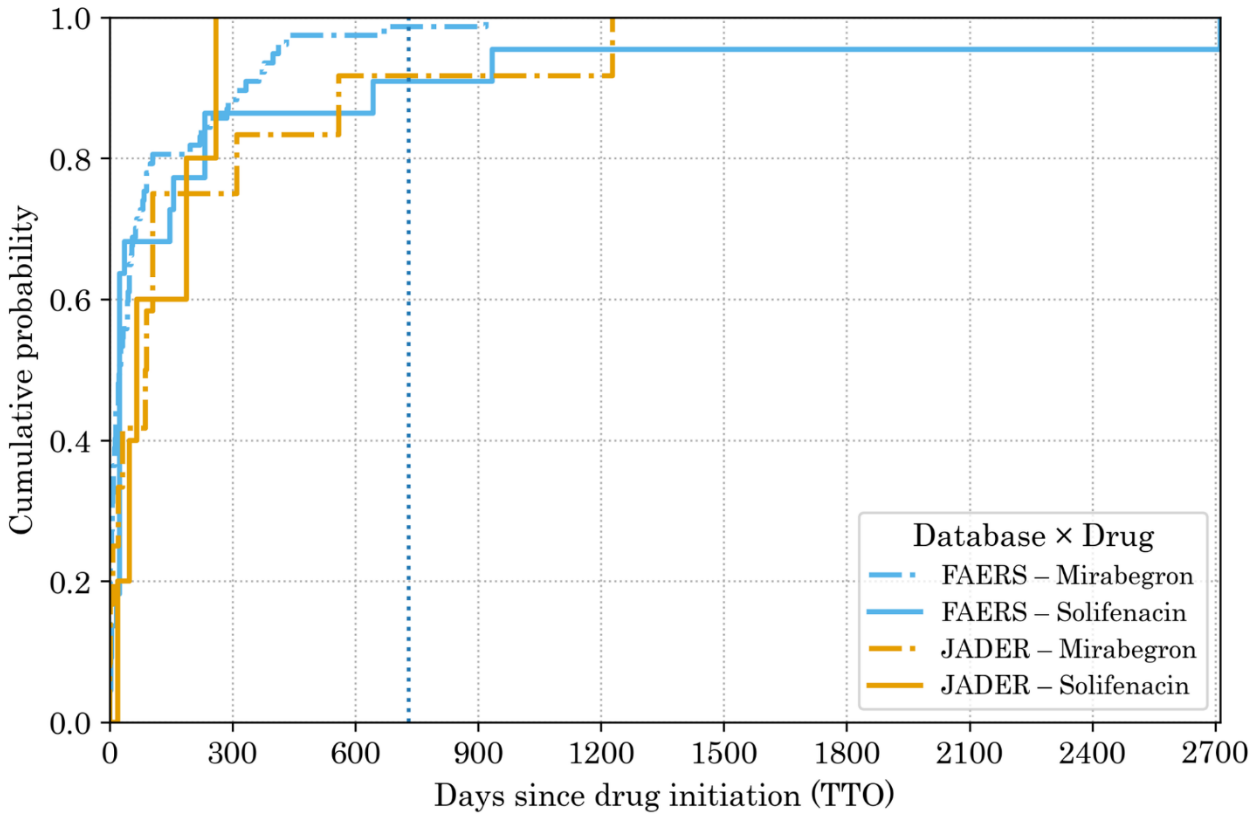

Supplementary Figure S4. Kaplan–Meier curves without the 2-year limit.

KM curves show the cumulative proportion of atrial fibrillation (AF) reports associated with solifenacin and mirabegron in JADER and FAERS over the full observation window (no 2-year truncation). Axes: x = time to onset (days); y = cumulative proportion of reports (%). Line styles: solid (solifenacin), dash–dot (mirabegron). Colors: JADER (orange), FAERS (blue). These descriptive visuals complement the main-text KM (≤2 years) by revealing late-onset reports and are not used for hypothesis testing.
